# Quality improvement in neonatal care through enhanced patient safety and clinical risk management: a before-and-after study about neonatal sepsis

**DOI:** 10.3389/fmed.2024.1430853

**Published:** 2024-08-20

**Authors:** Davide Ferorelli, Vito Maria Goffredo, Elena Graziano, Maurizio Mastrapasqua, Michele Telegrafo, Annachiara Vinci, Paolo Visci, Marcello Benevento, Fiorenza Zotti, Alessandra Foglianese, Raffaella Panza, Biagio Solarino, Alessandro Dell’Erba, Nicola Laforgia

**Affiliations:** ^1^Interdisciplinary Department of Medicine (DIM), Section of Legal Medicine, University of Bari “Aldo Moro”, Bari, Italy; ^2^Department of Interdisciplinary Medicine, Section of Neonatology and Neonatal Intensive Care Unit, University of Bari “A. Moro”, Bari, Italy

**Keywords:** neonatal sepsis, clinical risk management, patient safety, quality improvement, neonatal care

## Abstract

**Introduction:**

Neonatal sepsis, classified into early-onset and late-onset based on symptom timing, poses significant risks of morbidity and mortality, especially in low birth weight infants. Effective clinical risk management protocols are crucial in reducing these risks.

**Methods:**

This before-and-after study evaluated the impact of a newly implemented clinical risk management protocol in the Neonatology and Neonatal Intensive Care Unit (NICU) at Policlinico Hospital-University of Bari. The study included 399 neonates over three years, comparing pre- and post-protocol outcomes. Data collection focused on maternal and neonatal demographics, infection rates, and hospital stay lengths. Statistical analysis included *t*-tests, Wilcoxon-Mann–Whitney tests, and logistic regression models.

**Results:**

The study found no significant differences in neonatal pathologies or demographics between pre- and post-protocol groups. However, post-protocol implementation showed a notable reduction in umbilical venous catheter (UVC) infections (*p* = 0.018) and improved hospital stay lengths. Blood and urine cultures did not show significant changes in microbial patterns post-protocol.

**Discussion:**

The findings underscore the effectiveness of structured clinical risk management protocols in enhancing neonatal outcomes, particularly in reducing specific infection risks. Despite the study’s limitations, including its observational nature and sample size, the results advocate for broader adoption and further research on these protocols in diverse healthcare settings. The positive outcomes highlight the importance of continuous clinical risk management efforts in high-risk neonatal environments.

## Introduction

Sepsis is defined as a “clinical syndrome of potentially lethal organ dysfunction caused by a dysregulated response to infection” (1). Neonatal sepsis is a cause of morbidity and the third leading cause of neonatal mortality, especially in infants with low birth weight (Low Birth Weight < 2,500 g; Very Low Birth Weight < 1,500 g; Extremely Low Birth Weight < 1,000 g) (2). Neonatal sepsis can be classified into two major groups based on the timing of their presentation: Early-onset sepsis (EOS) occurs within 72 h of life, and Late-onset sepsis (LOS) occurs after 72 h of life (3). Epidemiological data about the EOS show an incidence of less than 1 per 1,000 live births when considering the entire neonatal population. However, the incidence is 10–15 per 1,000 live births among VLBW infants (4). The incidence of LOS is higher than that of EOS, especially among preterm infants, where it is estimated to be between 20–30 per 1,000 live births (3, 5).

Several factors increase the risk of neonatal sepsis, including: prolonged rupture of membranes, maternal infections, invasive procedures. Pathogens commonly involved in EOS are Group B Streptococcus (GBS), *Escherichia coli* (*E. coli*), *Listeria monocytogenes*, Other Gram-negative bacteria (Klebsiella, Pseudomonas, and Haemophilus) (6–8). Late-Onset Sepsis (LOS) The primary pathogens responsible for LOS include: Coagulase-negative Staphylococci (CONS), *Staphylococcus aureus*, Gram-negative bacteria (*E. coli*, Klebsiella, Enterobacter, and Pseudomonas), Fungal infections (Candida spp) (9, 10).

Several key issues in neonatal clinical risk have been described in the literature, including outdated pediatric guidelines, structural challenges, risks in departments like emergency and intensive care units, and the significant issue of drug administration errors (11). Therefore, clinical risk management tools were applied to this field in this study.

Indeed, our study underlines several key issues in pediatric clinical risk management, including the scarcity and outdated nature of pediatric guidelines, structural challenges in healthcare settings, the inherent risks of certain departments like emergency and intensive care units, and the prevalent issue of medication errors (12). Medication errors, particularly, are emphasized due to their frequency and the significant impact they have on patient safety. The discussion extends to strategies and tools for mitigating these risks and the integration of clinical pharmacists into intensive care units, showcasing their effectiveness in reducing errors (13).

Implementing procedures and protocols in clinical risk management is instrumental in reducing risks in clinical care, with various studies and analyses highlighting their effectiveness across different aspects of healthcare. According to S. Green et al. (14), such protocols provide essential training in risk management techniques for healthcare providers and establish agreed guidelines that contribute to lower rates of negligence claims and reduce malpractice insurance premiums in high-risk specialties (14).

Furthermore, the incorporation of risk management protocols into medical education promotes risk control habits among physicians, enabling them to practice quality medicine with reduced concerns for malpractice reprisal and peer review (10). M. Gulino et al. emphasize that these protocols introduce prevention and management instruments that contribute to error reduction and quality enhancement in healthcare services (15). This sentiment is echoed by J. Samanta and A. Samanta, who highlight the role of clinical guidelines linked to the medical evidence base in enhancing care quality and minimizing treatment variations, ultimately preventing harm to patients (16).

Clinical risk management shifts the focus from litigation protection to caring for injured patients and meeting their needs, thereby improving the quality of care and reducing harm (17). R. Clements also points out the goal of decreasing adverse events and harm to patients, minimizing claims, and managing claim costs effectively through continuous improvement focused on patient welfare (18).

A protocol has been implemented in the Neonatology and Neonatal Intensive Care Unit (NICU) of the Policlinico Hospital-University of Bari to prevent healthcare-associated infections, ventilator-associated pneumonia, central line bloodstream infections (CLABSI), and epidemic events.

This study aims to evaluate whether, following the introduction of the protocol described, there is an improvement in the outcomes of neonates admitted to the Neonatology and Neonatal Intensive Care Unit (NICU).

### Study objectives

The objective of this study is to assess whether, following the introduction of the protocol described in the previous chapter, there is an improvement in the outcomes of neonates admitted to the Neonatology and Neonatal Intensive Care Unit (NICU).

## Materials and methods

**Figure 1:**
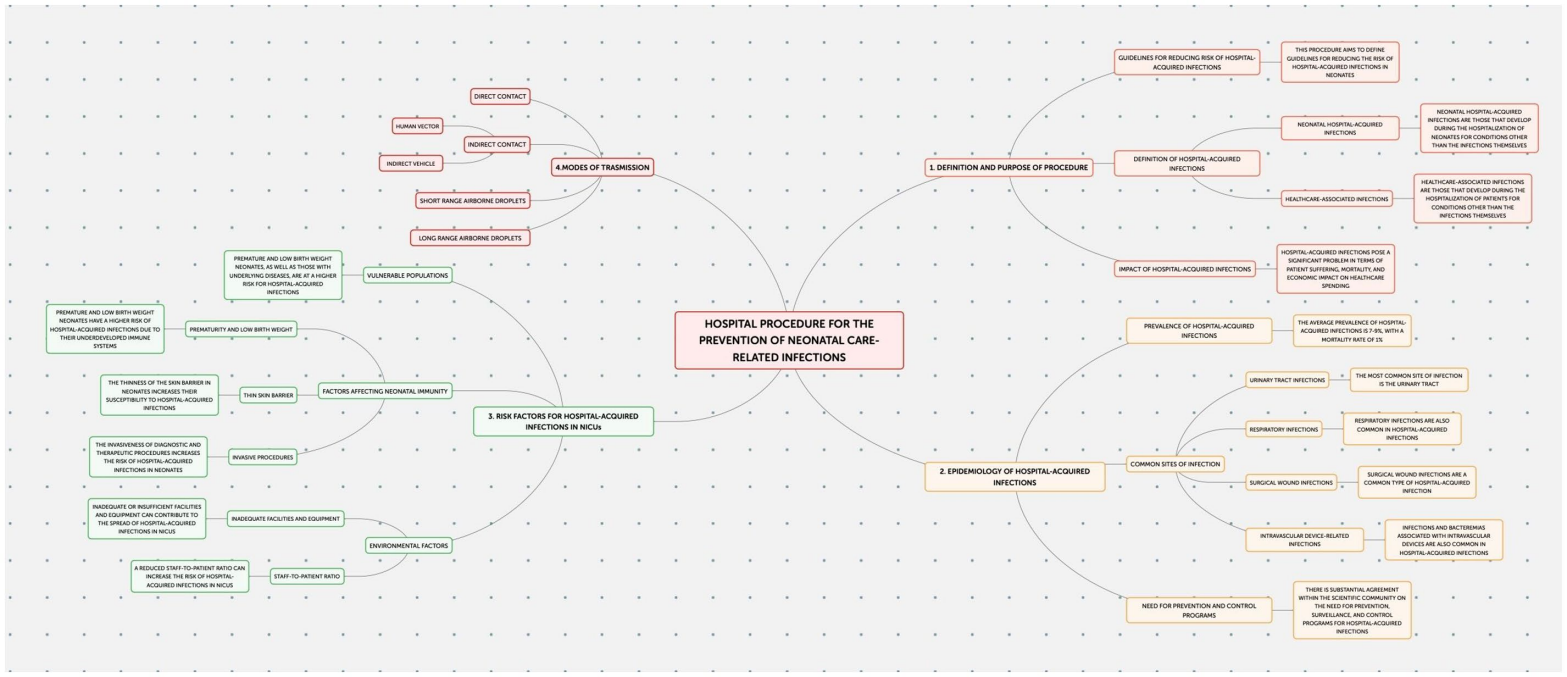
Hospital procedure for the prevention of neonatal care-related infections.

**Figure 2:**
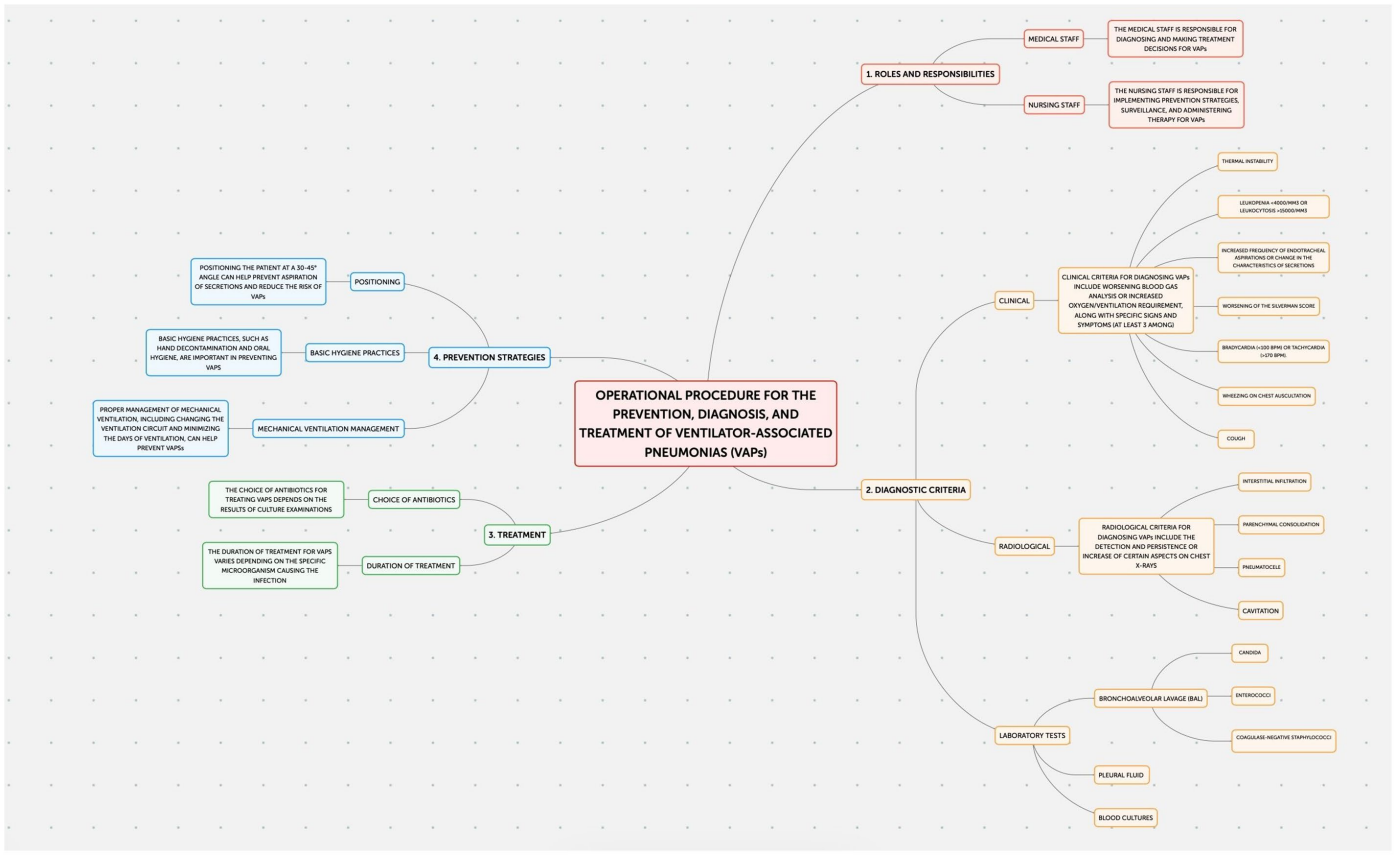
Operational procedure for the prevention, diagnosis, and treatment of ventilator-associated pneumonias (VAPs).

**Figure 3:**
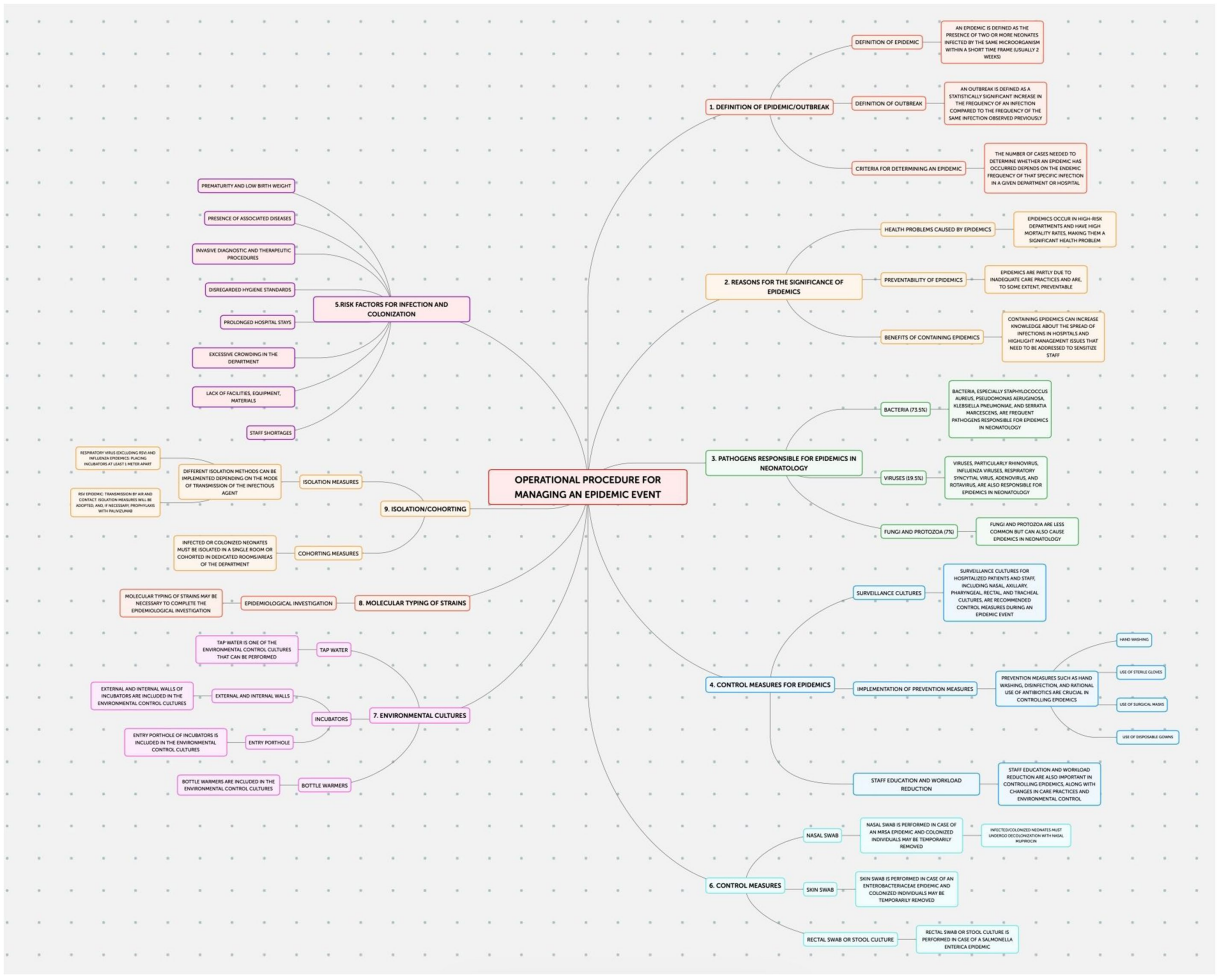
Operational procedure for the prevention of infections associated with intravascular devices.

**Figure 4:**
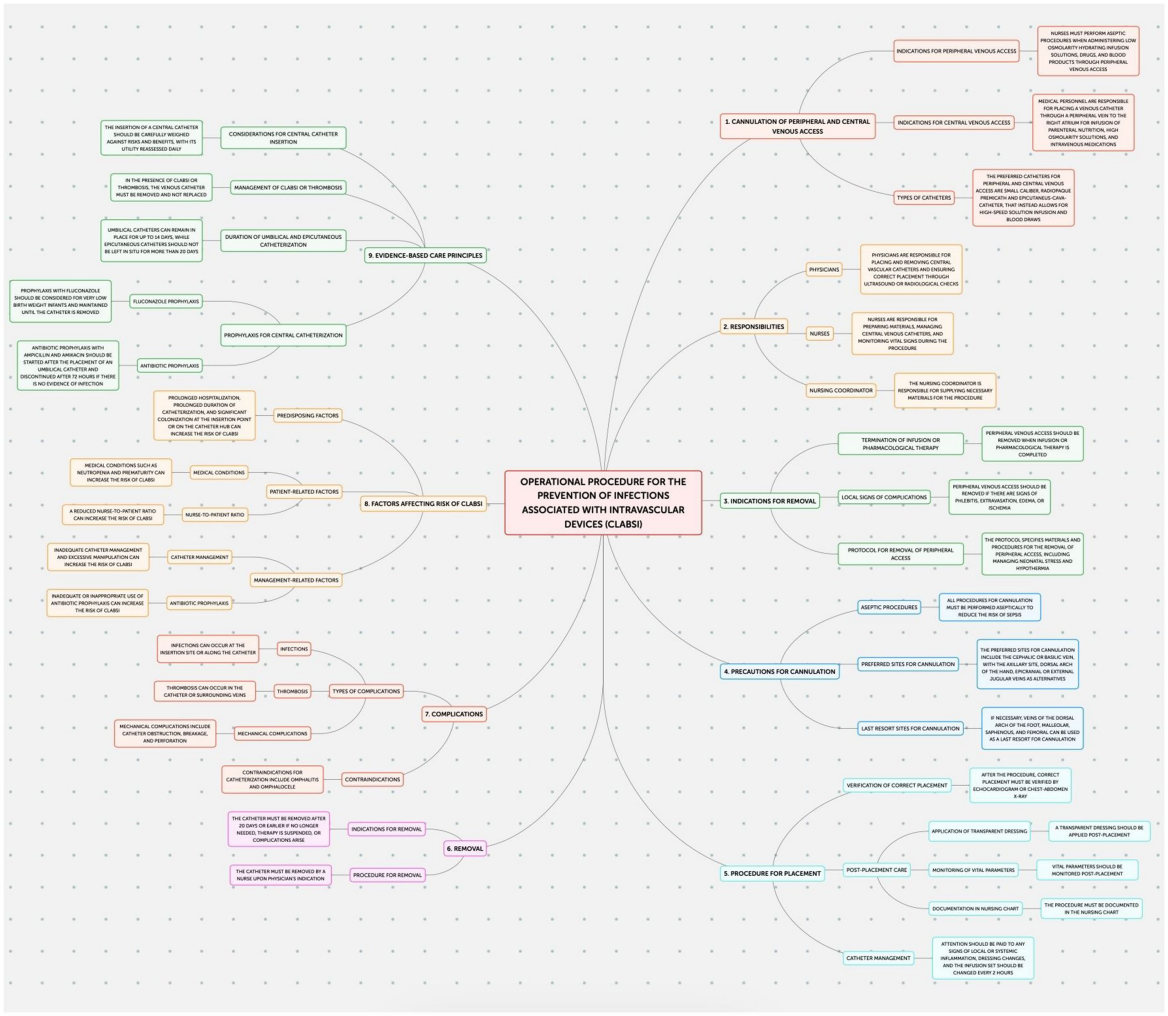
Operational procedure for managing an epidemic event.

This study employed an observational design to examine neonatal outcomes at the Policlinico Hospital-University of Bari, a tertiary care facility with over 1,000 beds, after protocol introduction. The protocol, implemented in the Neonatology and Neonatal Intensive Care Unit (NICU) of the Policlinico Hospital-University of Bari, consists of four documents addressing the prevention of: Healthcare-Associated Infections (HAIs) (Figure 1); Ventilator-Associated Pneumonias (VAPs) (Figure 2); central line bloodstream infections (CLABSI) (Figure 3); and epidemic events (Figure 4). The protocol applies to two areas of Neonatology and NICU (Neonatal Intensive Care and Sub-intensive Care) and is directed at all staff working in these areas (both regularly and occasionally) as well as the families of hospitalized infants.

The STtrengthening the Reporting of OBservational studies in Epidemiology (STROBE) guidelines for reporting of observational studies were followed. The research protocol was approved by the local General Health Directorate (nr. 1,497 on 2020.11.19).

All patients admitted to the Neonatology and NICU of the Policlinico Hospital-University of Bari in three-year period 1 January 2019–31 December 2021 were enrolled.

The data collection period spanned from January 2019 to December 2021, encompassing the COVID-19 pandemic. This period saw an increase in healthcare-associated infections, including superinfections and co-infections, which could influence neonatal outcomes. However, none of the newborn patients in our sample, whether born to COVID-positive or COVID-negative mothers, tested positive for COVID-19 (19).

For each participant, both maternal and neonatal demographic and anamnestic data regarding pregnancy, delivery and the postnatal period were systematically collected from medical charts. Data regarding neonatal urine and blood cultures, swabs, infections (i.e., sepsis; central venous catheter (CVC), peripheral venous catheter (PVC), and umbilical venous catheter (UVC) infections) and antibiotic therapy were specifically collected.

Neonates were classified as preterm if born at gestational ages <37 weeks and as low birth weight if weighing <2,500 g. Data anonymization was ensured through the use of coded keys.

Based on the approval date of protocol, participants were categorized into “pre” and “post” the introduction of the protocol in July 2020.

Microsoft Excel software was utilized for data collection, and Jamovi-Electron software was used for statistical processing.

Quantitative variables were reported as mean, standard deviation, and interquartile ranges. The normality of distribution was assessed using Q-Q plots, skewness, kurtosis, and the Shapiro–Wilk test. The *t*-Student test was applied to parametric quantitative variables, and the Wilcoxon-Mann–Whitney test was used for non-parametric ones. Qualitative variables were presented in proportions, and their distribution was analyzed using the χ^2^-test with Fisher’s correction as needed (for group sizes <5 units). Odds Ratio (OR) with 95% confidence intervals (CI95%) was calculated using logistic regression models as a measure of association. Results with *p* < 0.05 were considered statistically significant. Correlation between variables was assessed using the Pearson Test, with significance set at *p* > 0.05.

## Results

Our analysis encompassed a cohort of 399 neonates, segregated into pre-protocol (39.2%, *n* = 156) and post-protocol (60.8%, *n* = 243) groups, aiming to evaluate the efficacy of a newly implemented neonatal care protocol. Sex distribution within the cohort was similar pre- and post-protocol, with 40.45% females (*n* = 161) and 59.55% males (*n* = 237), showing no significant sex-based disparities in protocol outcomes (χ^2^-test, *p* > 0.05).

Mean gestational age was 34.5 (SD 4.40) weeks for the pre-protocol group and 35.0 (SD 4.40) weeks for the post-protocol group, with distribution analyses indicating Gaussian curves for both (Shapiro–Wilk test, *p* < 0.001), yet no statistically significant difference was identified between the groups (*t*-Student test, *p* = 0.128).

Mean birth weight was 2,438 g (SD 947) in the pre-protocol group and 2,289 g (SD 948) in the post-protocol group, both with normal distribution (Shapiro–Wilk test, *p* < 0.01 for post; *p* = 0.001 for pre) and no significant difference in birth weight across groups (*t*-Student test, *p* = 0.214). Birth weight categories (>2,500 g vs. <2,500 g) were similar between pre- and post-protocol groups (χ^2^ test, *p* > 0.05).

Delivery methods (spontaneous vs. cesarean) and the incidence of multiple gestation did not differ between pre and post protocol groups (χ^2^-test, *p* > 0.05), as well as the ratio between outborn and inborn (χ^2^-test, *p* > 0.05).

**Figure 5:**
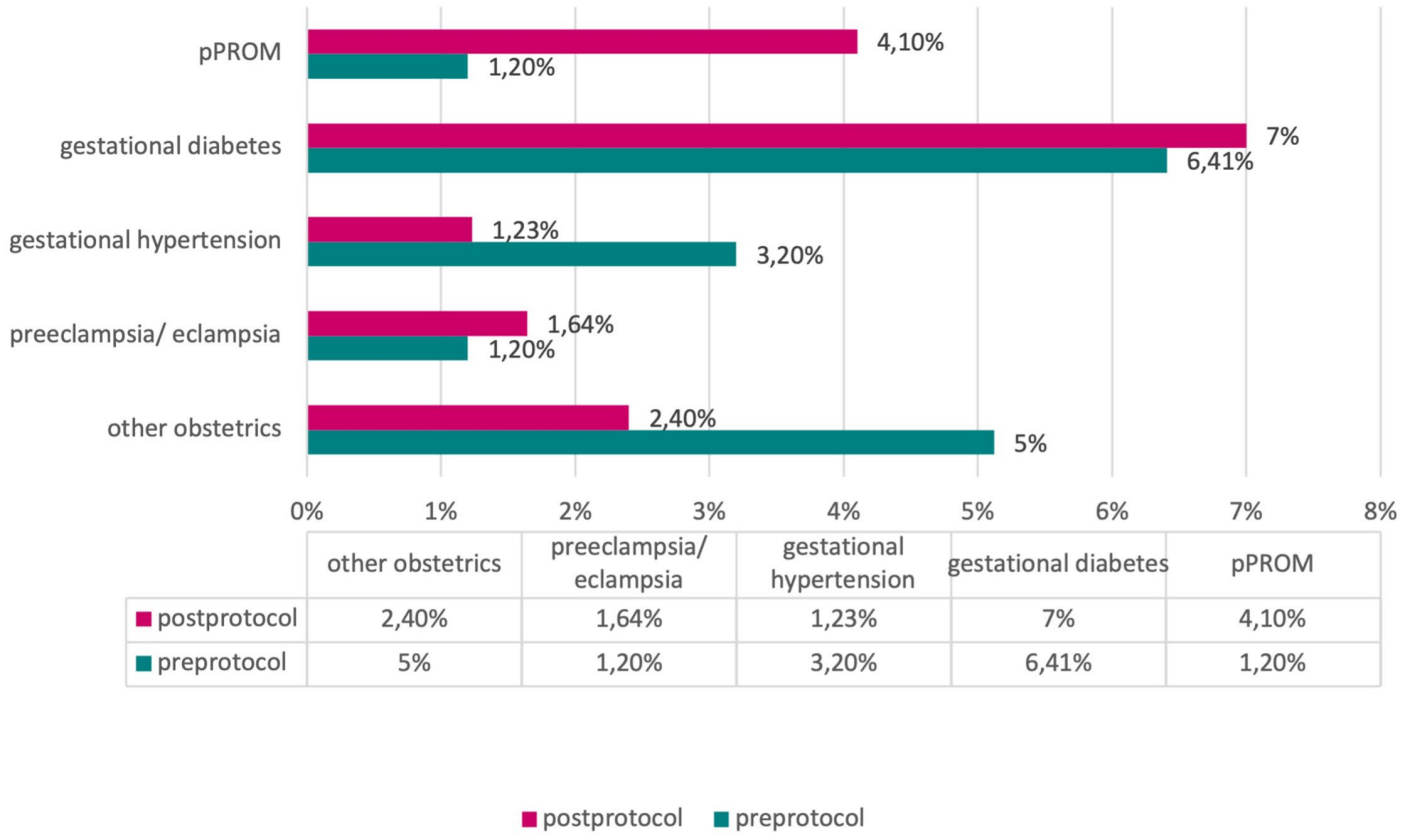
Frequency of obstetric pathologies in the ‘pre’ and ‘post’ protocol groups.

In assessing maternal pathologies, including gestational diabetes, gestational hypertension, preeclampsia/eclampsia, preterm premature rupture of membranes (pPROM), and other obstetric pathologies, our analyses did not reveal significant differences between the pre- and post-protocol groups (χ^2^-test, *p* > 0.05) (Figure 5).

Neonatal pathologies and diagnoses upon admission did not exhibit statistically significant variances, suggesting the protocol’s effectiveness transcends specific neonatal health conditions (χ^2^-test, *p* > 0.05) (Tables 1, 2).

**Table 1:** Contingency table for the “admission diagnosis” variable.

Admission diagnosis	Period		Total	*p*-value
	post	pre		
Others	97	66	163	
Cardiovascular	12	3	15	
Gastrointestinal	24	8	32	
Infectious	6	3	9	
Malformative	25	13	38	
Metabolic	7	6	13	
Neurologic	5	4	9	
Obstetric	2	3	5	
pPROM	10	2	12	
Respiratory	54	46	100	
Total	242	154	396	0.246

**Table 2:** Contingency table for the “neonatal pathologies” variable.

Neonatal pathology	Period		Total	*p* value
	post	pre		
Others	38	13	51	
Cardiovascular	11	6	17	
Gastrointestinal	17	5	22	
Genitourinary	3	2	5	
Infectious	28	18	46	
Malformative	23	16	39	
Metabolic	11	6	17	
Neurologic	7	5	12	
Ophtalmic	5	3	8	
Respiratory	97	79	176	
Total	240	153	393	0.415

The microorganisms responsible for infections associated with umbilical (UVC), central (CVC), and peripheral (PVC) catheters infections were categorized as follows: Fungi: Candida. Gram-positive: Enterococcus; Staphylococcus. Gram-negative: Enterobacter; *E. coli*; Serratia. Gram-positive and Gram-negative: Staphylococcus and Klebsiella; Staphylococcus and Serratia; Staphylococcus and Pseudomonas. These findings are consistent with those reported in the literature (20).

**Figure 6:**
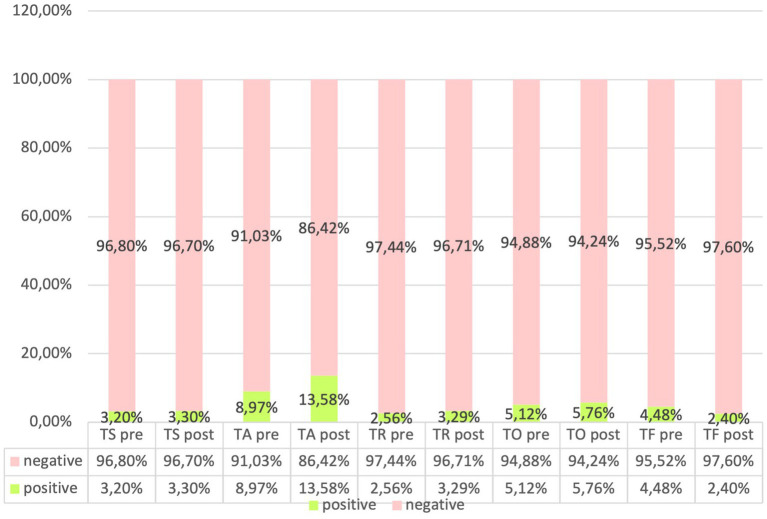
Frequency of swab positivity in the ‘pre’ and ‘post’ groups.

**Figure 7:**
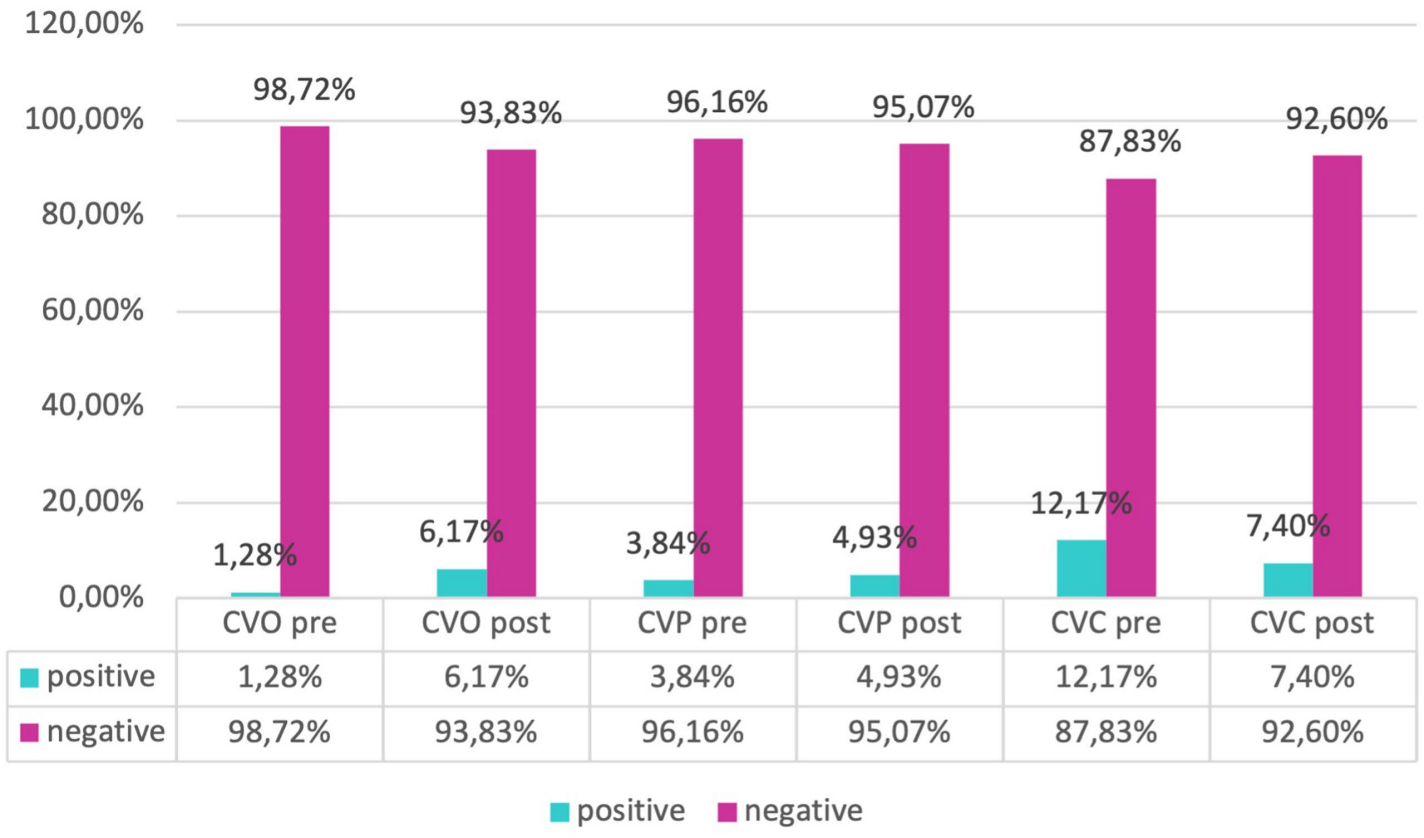
Frequency of positivity and negativity of umbilical, peripheral, and central catheters.

Our evaluation of catheter infections showed no significant changes of positive cultures both for central and peripheral venous catheters between pre and post-protocol implementation, but a marked reduction of infections of umbilical venous catheter (UVC) infections (χ^2^-test, *p* = 0.018) was found, indicating a targeted effect of the protocol on reducing specific infection risks (Figures 6, 7).

In the “post” group, 10.25% (n = 25) of the blood cultures were positive, while 89.75% were negative. In the “pre” group, 7.70% (n = 12) were positive, and 92.30% (n = 144) were negative. The isolated microorganisms were categorized as follows: Gram-positive and Fungi: Enterococcus and Candida; Staphylococcus and Candida. Gram-positive: Bacillus; Corynebacterium; Staphylococcus; Streptococcus. Gram-negative: Enterobacter; *E. coli*; Klebsiella; Serratia. Gram-positive and Gram-negative: Staphylococcus and Klebsiella. These findings are consistent with those reported in the literature (21).

The microorganisms from urine cultures were grouped as follows: Gram-negative and Gram-negative: Klebsiella and Enterobacter; Klebsiella and *E. coli*; Klebsiella and Morganella; Pseudomonas and Klebsiella. Gram-negative and Gram-positive: *E. coli* and *Enterococcus faecalis*; Enterococcus and Escherichia; Klebsiella and Enterococcus; Proteus and Enterococcus; Pseudomonas and Enterococcus. Gram-negative: Enterobacter; Escherichia; Klebsiella; Pseudomonas; Serratia. Gram-positive: Enterococcus; Streptococcus. Gram-negative and Fungi: Escherichia and Candida.

The microorganisms responsible for positive swab results from various sites (surface, ear, rectal, ocular, and pharyngeal) were categorized as follows: Viruses: Respiratory Syncytial Virus. Gram-positive: Staphylococcus; Enterococcus; Streptococcus; *Enterococcus faecalis*. Gram-negative: Morganella; Pseudomonas; *E. coli*; Campylobacter; Klebsiella; Enterobacter; Proteus; Serratia; Haemophilus. Gram-positive and Gram-positive: Staphylococcus and Enterococcus. Gram-positive and Gram-negative: Staphylococcus and Pseudomonas; Streptococcus and Klebsiella; Staphylococcus and *E. coli*; Staphylococcus and Klebsiella. Gram-positive and Fungi: Streptococcus and Candida.

All these findings are consistent with those reported in the literature (22–24).

**Figure 8:**
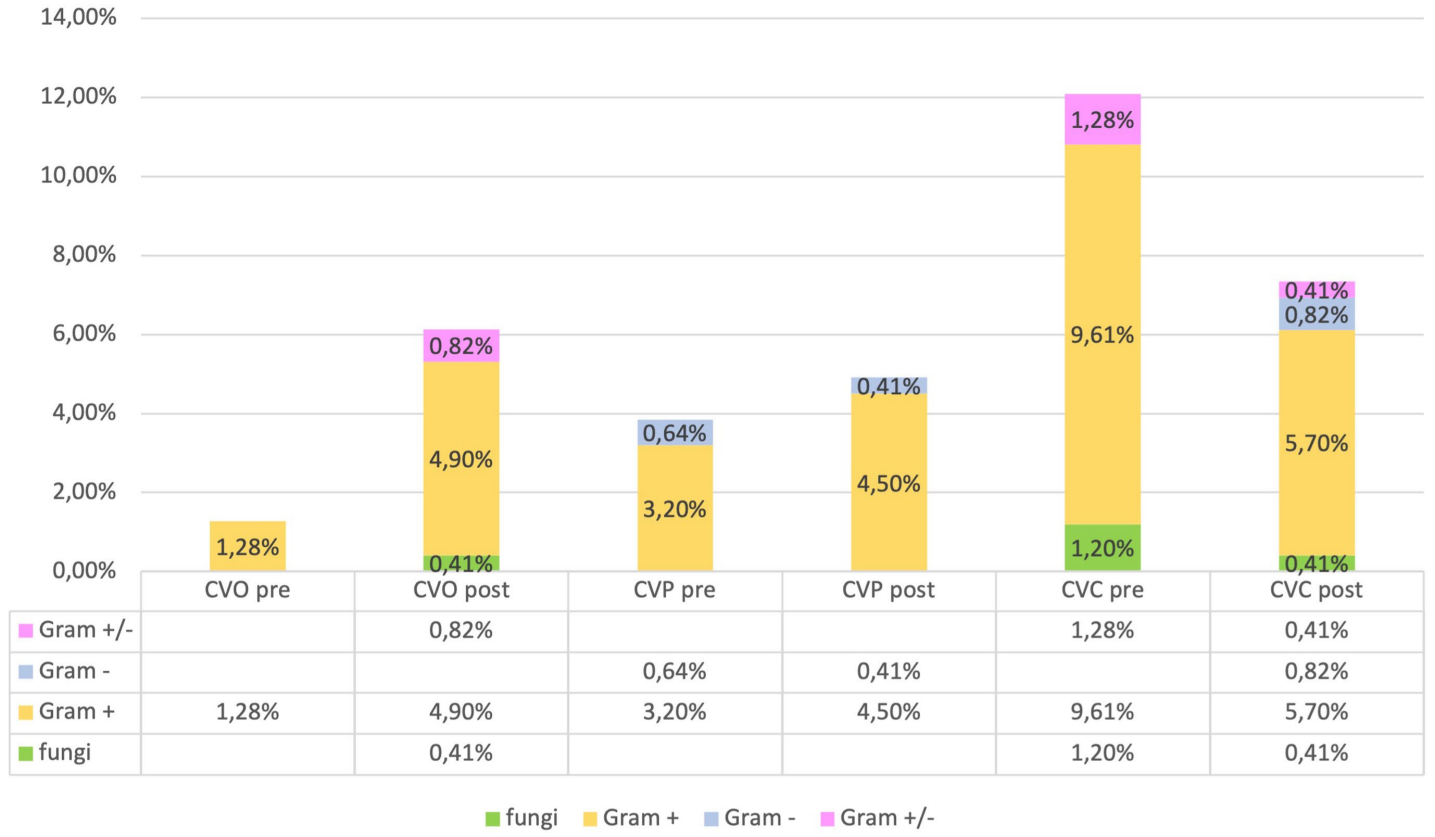
Frequency of CVC, CVO, CVP infections in the ‘pre’ and ‘post’ groups.

Analyzing microbial involvement in swab positivity and culture outcomes, we found no significant alterations in microbial patterns post-protocol, reinforcing the protocol’s effectiveness in maintaining a stable microbial environment within the NICU (χ^2^-test, *p* > 0.05) (Figure 8).

Notably, our inferential statistical analysis highlighted significant associations between gestational age and neonatal outcomes, with gestational age showing a significant correlation with birth weight (Pearson test, *p* < 0.05) and inversely with hospital stay and therapy duration. Logistic regression unveiled a statistically significant improvement in outcomes related to CVC infections post-protocol (*p* = 0.012, OR = 0.1912), underscoring the protocol’s success in enhancing neonatal care within the NICU setting (Table 3).

**Table 3:** Logistic regression model aimed at investigating the effects of the protocol on the measured clinical outcomes.

Predictor	Estimate	SE	Z	*p*	Odds ratio	95% Confidence Interval	
						Lower	Upper
Intercept	7.43458	3.3560	2.2153	0.027	1693.5428	2.3559	1.22e+6
UVC qual							
Yes – no	1.39212	1.2156	1.1453	0.252	4.0234	0.3715	43.578
PVC qual							
Yes – no	−0.08663	0.8802	−0.0984	0.922	0.9170	0.1634	5.148
CVC qual							
Yes – no	−1.65441	0.6554	−2.5242	0.012	0.1912	0.0529	0.691
Surface qual							
Yes – no	1.27359	0.9336	1.3642	0.172	3.5736	0.5734	22.272
Auricular qual							
Yes – no	0.76105	0.5925	1.2844	0.199	2.1405	0.6701	6.837
Rectal qual							
Yes – no	−0.13721	1.3035	−0.1053	0.916	0.8718	0.0677	11.218
Ophtalmical qual							
Yes – no	−0.56205	1.1068	−0.5078	0.612	0.5700	0.0651	4.989
Pharyngeal qual							
Yes – no	−3.31497	2.1047	−1.5750	0.115	0.0363	5.87e-4	2.248
Urine qual							
Yes – no	−0.58876	0.6659	−0.8841	0.377	0.5550	0.1505	2.047
EMO qual							
Yes – no	0.24158	0.9194	0.2628	0.793	1.2733	0.2100	7.718
Sepsis							
Yes – no	0.26171	0.7608	0.3440	0.731	1.2991	0.2925	5.771
Weight	1.65e-4	4.08e-4	0.4056	0.685	1.0002	0.9994	1.001
Recovery days	0.03182	0.0153	2.0792	0.038	1.0323	1.0018	1.064
Gestational age	−0.20336	0.1027	−1.9810	0.048	0.8160	0.6673	0.998
DOT (days of therapy)	−0.04500	0.0763	−0.5896	0.555	0.9560	0.8232	1.110
APGAR 1 min	−0.07252	0.1708	−0.4246	0.671	0.9300	0.6655	1.300
APGAR 5 min	−0.00989	0.2405	−0.0411	0.967	0.9902	0.6180	1.586

Estimates represent the log odds of “PERIOD = post” vs. “PERIOD = pre”.

## Discussion

The current study offers an extensive evaluation of the effects of a newly implemented protocol on neonatal sepsis prevention outcomes. The sample comprised 399 neonates, segmented into pre-protocol (39.2%) and post-protocol (60.8%) cohorts. This segmentation facilitated a thorough comparison of outcomes prior to and subsequent to the protocol’s introduction, thereby aiming to determine its efficacy in enhancing neonatal care, consistent with the existing scientific literature (25, 26).

The two samples are homogeneous for variables such as sex (40.45% females, 59.55% males), mean gestational age (pre 34.5 weeks, post 35.0), mean birth weight (pre 2,438 g, post 2,289 g), normal weight/underweight, in/outborn, multiple gestation, type of delivery, maternal diseases (obstetric and non-obstetric), diagnosis upon admission to neonatology, and neonatal pathology. This has an undeniable advantage, as it allows for the comparison of two groups that can be considered homogeneous.

Consequently, observed variations in outcomes can be attributed with greater confidence to the effects of the intervention protocol rather than to underlying demographic or clinical disparities, in accordance with scientific evidence related to the prevention of sepsis in adults (27–30). The analysis of infections related to central venous catheters (CVC) and peripheral venous catheters (PVC) is crucial for assessing the relationship between the concentration of microorganisms and the positivity of the culture (31). Regarding infections associated with umbilical venous catheters (UVC), which are commonly inserted in neonates for vascular access and are not without complications (32), studies with similar samples have reported an incidence of related septicemia of 9.5% (33). Despite this, the use of UVC remains the standard of care in the neonatal intensive care unit (NICU) for administering fluids, medications, and parenteral nutrition (34). Our evaluation of catheter-related infections revealed no significant differences in the rate of positive cultures for both central and peripheral venous catheters following the implementation of the protocol. However, a notable reduction in umbilical venous catheter (UVC) infections was observed, suggesting that the protocol effectively targeted specific infection risks. The NICU environment, colonized by various microorganisms, enhances the risk of developing antibiotic resistance (35). Furthermore, an increase in antibiotic-resistant organisms could lead to a rise in neonatal case fatality rates, underscoring the necessity for regular surveillance (36, 37). Analysis of microbial involvement in swab positivity and culture outcomes revealed no significant changes in microbial patterns post-protocol, supporting the protocol’s effectiveness in maintaining a stable microbial environment within the NICU. Inferential statistical analysis showed significant associations between gestational age and neonatal outcomes, consistent with existing literature (38, 39). Specifically, gestational age was significantly correlated with birth weight and inversely correlated with the duration of hospital stay and therapy. Logistic regression analysis indicated a statistically significant improvement in outcomes related to central venous catheter (CVC) infections post-protocol, highlighting the protocol’s success in enhancing neonatal care within the NICU.

This demonstrates that proactive application of protocols aimed at improving patient safety is a virtuous activity that can also prevent neonatal sepsis (40, 41). Thus, it can be affirmed that the introduced protocol effectively reduces infection risks in neonates admitted to the NICU, critically impacting patient safety, hospital costs, and overall quality of care.

The absence of statistically significant differences in other analyzed variables, such as neonatal and maternal diseases and the type of delivery, further supports the conclusion that the observed improvements in neonatal outcomes are attributable to the protocol. The proven effectiveness of the introduced protocol allows for two considerations. Firstly, the observed results align with the objectives of the study, enhancing outcomes for hospitalized neonates, which inevitably affects costs and the entire care pathway. Secondly, consistent with international literature, clinical risk management and preventive measures are effective (42–44).

Despite these promising results, the study recognizes limitations related to sample size and suggests the potential value of repeating the study with a larger sample and possibly in a multicentric setting to provide further validation of the protocol’s effectiveness and refine its components for even greater impact on neonatal care.

This study underscores the positive impact of a targeted protocol on reducing the risk of CVC infections and the length of hospital stays in a neonatal intensive care setting. These findings emphasize the importance of evidence-based interventions in improving neonatal outcomes (45) and highlight the ongoing need for research to optimize care protocols in high-risk healthcare environments (46).

One of the major challenges in applying the protocol was the lack of comprehensive anamnestic data, which made it difficult to fully assess risk factors. Additionally, the resistance to antibiotics observed in some cases highlights the need for ongoing monitoring and adjustment of treatment protocols.

In the context of our epidemiological investigations within the neonatal intensive care unit (NICU), a specific report on neonatal nosocomial infections was not made. Consequently, there is no documented trend of nosocomial infections in the involved Department.

However, to address this lack of direct data, we used the number of umbilical venous catheter (UVC) infections as a proxy indicator. The use of UVCs is common in neonatal intensive care units for administering medications, fluids, and parenteral nutrition to premature or critically ill infants. Since infections related to UVCs represent a significant proportion of nosocomial infections in these units, their number can be considered a useful indicator to indirectly monitor the prevalence of neonatal nosocomial infections.

We collected data related to UVC infections through the NICU’s internal recording system, analyzing the period between 2019 and 2021. These data provide an indicative picture of the trend of nosocomial infections and allow us to implement targeted control and prevention strategies.

Despite this limitation, the use of UVC infection as a proxy offers us a critical insight into current practices and areas needing improvement, thereby contributing to the overall quality of neonatal care in our Department.

## Conclusion

This study assessed a clinical risk management protocol implemented in the Neonatology and Neonatal Intensive Care Unit at Policlinico Hospital-University of Bari, involving 399 neonates. The protocol was designed to improve patient safety by mitigating healthcare-associated infections and other adverse events. Results indicated a significant decrease in central venous catheter infections and shorter hospital stays post-implementation, highlighting the protocol’s effectiveness in enhancing neonatal outcomes and healthcare efficiency.

Despite its observational nature, the study underscores the significance of structured clinical risk management in neonatology. It posits that targeted preventive measures and staff training can substantially diminish risks associated with neonatal care. Future research involving a larger, multicentric sample is advised to further corroborate these findings and assess the protocol’s adaptability across various settings, especially given that nosocomial infections are critically linked to medical liability, escalating healthcare costs, and diminishing public trust in healthcare systems (41–47).

In conclusion, the protocol shows promise as a blueprint for improving care standards in NICUs, advocating its wider adoption to establish new benchmarks in the quality and safety of neonatal care.

## Data availability statement

The datasets presented in this article are not readily available because privacy. Requests to access the datasets should be directed to paolo.visci@uniba.it.

## Ethics statement

The studies involving humans were approved by General Health Directorate (nr. 1497 on 2020.11.19). The studies were conducted in accordance with the local legislation and institutional requirements. Written informed consent for participation was not required from the participants or the participants’ legal guardians/next of kin in accordance with the national legislation and institutional requirements.

## Author contributions

DF: Writing – original draft, Writing – review & editing. VG: Writing – original draft, Writing – review & editing. EG: Writing – original draft, Writing – review & editing. MM: Writing – original draft, Writing – review & editing. MT: Writing – original draft, Writing – review & editing. AV: Writing – original draft, Writing – review & editing. PV: Writing – original draft, Writing – review & editing. MB: Writing – original draft, Writing – review & editing. FZ: Writing – original draft, Writing – review & editing. AF: Writing – original draft, Writing – review & editing. RP: Writing – original draft, Writing – review & editing. BS: Writing – original draft, Writing – review & editing. AD'E: Writing – original draft, Writing – review & editing. NL: Writing – original draft, Writing – review & editing.
